# Screening for infectious diseases in newly arrived migrants in Europe: the context matters

**DOI:** 10.2807/1560-7917.ES.2018.23.28.1800283

**Published:** 2018-07-12

**Authors:** Takis Panagiotopoulos

**Affiliations:** 1 European Centre for Disease Prevention and Control (ECDC), Stockholm, Sweden

**Keywords:** migrant health, screening, tuberculosis, enteric infections, schistosomiasis, strongyloidiasis

In the past three decades there has been a considerable increase in the number of migrants globally. In 2015, about one third of the world’s migrants lived in Europe (ca 75 million, which is about 10% of the area’s population), contributing to the region’s economy and creating a younger demographic composition [1,2]. In recent years, an unprecedented number of forcibly displaced persons fleeing conflict, violence or disaster have come to Europe. In 2015, the peak year of this wave, more than one million asylum seekers, refugees and irregular migrants arrived in Europe [3]. For the purpose of this editorial, economic immigrants, asylum seekers and refugees are collectively referred to herein as migrants.

The *Eurosurveillance* series on screening for infectious diseases in newly arrived migrants in Europe is well timed. Seven articles are included in this series: two systematic reviews on the effectiveness and cost-effectiveness of screening for active tuberculosis (TB) and for latent TB, and five articles presenting the experiences of screening programmes—for active and latent TB, hepatitis B, hepatitis C, HIV infection, and infection with selected enteric bacteria (e.g. *Salmonella* spp., *Shigella* spp.) and helminths (e.g. *Schistosoma* spp., *Strongyloides stercoralis,* *Ascaris* *lumbricoides*)—for migrants who arrived recently in several parts of Europe [4-10].

Since 2000, TB has consistently decreased in the European Union (EU) and European Economic Area (EEA). However, the current rate of decrease is insufficient to achieve the End TB Strategy targets [11-13]. In 2016, one third of all new active TB cases reported in EU/EEA countries were diagnosed in people who were born outside of the country where the case was reported or who had foreign citizenship [11]. Policies to promote timely diagnosis and treatment in migrants are, therefore, crucial [14].

The systematic review by Greenaway et al., on the effectiveness and cost-effectiveness of screening for active TB in migrants using chest X-ray as the screening test, demonstrates the heterogeneity of yield among screening programmes, which reflects the heterogeneity of disease prevalence [4]. The authors did not identify any study on the effectiveness of a screening programme as a whole and, therefore, studies on yield, sensitivity and specificity of chest X-ray to detect active TB, effectiveness of treatment and uptake of screening were reviewed. As expected, yield tends to be higher in migrant populations originating from countries with a higher incidence of endemic TB. Yield was also shown to depend on the cause for migration and the setting in which screening was carried out, a parameter that probably reflects living conditions, migration routes and migration experience. Chest X-ray was found highly sensitive but only moderately specific, and its acceptance by migrants was generally good. The authors point out that although screening for active TB would be more efficient if targeted to migrants from high TB incidence countries, many cases occur in migrants from countries with lower TB incidence and the heterogeneity between different locations in Europe limits the ability to make precise recommendations. They therefore underline that policies should be tailored to the local epidemiology of TB and emphasise the importance of addressing the issue of barriers to treatment and care for all migrants.

The latter conclusion is in line with the results of the article by Kuehne et al., who found poorer treatment outcomes in cases of pulmonary TB identified through screening in newly arrived asylum seekers in Germany from 2002–2014, compared with cases identified in other ways (diagnosis of symptomatic patients, identification of cases through contact tracing) [6]. The authors concluded that ‘finding and losing’ should be avoided by linking migrants with positive screening results to treatment facilities and by investigating possible barriers to treatment completion.

The systematic review by Greenaway et al. on screening migrants for latent TB infection (LTBI) points out the importance of this issue, as the majority of TB cases in migrants in the EU/EEA are due to reactivation of LTBI [5]. Nevertheless, there is an inherent limitation in any screening policy for LTBI: currently available tests cannot distinguish the 5–15% of LTBI cases who will progress to active TB and may therefore benefit from treatment [5,15]. The review summarises evidence that groups at highest risk for progression from LTBI to active TB include people with immunosuppressive conditions (e.g. HIV infection), those who were infected recently, migrants from endemic countries with high TB incidence and those who have experienced crowded living conditions and perilous journeys. Sequential tuberculin skin testing and interferon-gamma release assay was generally found more cost-effective than single testing with either of the two tests. Barriers at patient, provider and structural levels may result in loss to follow-up and jeopardise treatment completion of eligible patients. The authors concluded that migrant-focused LTBI screening programmes may be effective and cost-effective if they are highly targeted and ensure high screening uptake, health care access and treatment completion. Further, the findings of Mueller-Hermelink et al. highlight that migrant children under the age of 6 years are at higher risk for progression from LTBI to active TB compared to older migrant children or adolescents, and effective options of prophylactic treatment are available [8,16].

Two studies in this series report findings that include stool screening for helminthic infections in Italy and in Germany [9,10]. They confirm the conclusion of previous studies that the frequency of positive screening test results depends on migrants’ country of origin [17]. The Italian study found positive stool results for *Schistosoma mansoni* eggs in 7.0% of 270 migrants from sub-Saharan Africa and none in 79 screened migrants from Asia; in the German study, 0.3% of 14,511 individuals originating from a variety of countries had positive stools for *Schistosoma mansoni* eggs.*

These studies also concur in another important finding: they confirm that possible enteric infections in migrants do not spill over into the local population at any appreciable degree. In particular, the German investigators addressed this issue by documenting that during the study period they did not identify any records of secondary transmission of *Salmonella* spp. or *Shigella* spp. to the host population [10]. Moreover, the studies agree that the rationale for screening migrants for enteric pathogens is mainly to prevent severe morbidity in infected individuals [9,10]. Diseases that can remain asymptomatic for a long time and lead to chronic infection with severe sequelae, like some helminthic infections, could therefore be candidates for screening [18].

The studies by Bil et al. and Buonfrate et al. support the feasibility of combined preventive programmes for newly arrived migrants in some settings, including screening for hepatitis B, hepatitis C and HIV infection, but they also show that serological evidence of infection can differ greatly between programmes and migrant groups [7,9].

A common finding of the articles in the present series corroborates a major conclusion of previous studies and reviews: migrants do not represent a significant risk for EU/EEA populations in terms of infectious disease incidence in the local population and infectious disease outbreaks [19,20]. The series adds substantial evidence to the existing body of knowledge about this in relation to TB, as well as to bacterial and helminthic enteric infections [4-10]. Despite general agreement in the scientific community, the issue continues to be debated controversially in several European countries [21]. Clear communication of existing evidence on this topic is, therefore, a priority.

A further common element in many of the articles in this series is that, despite existing limitations, potentially effective screening tools for several infectious diseases do exist, but making general recommendations for universal use is not supported by evidence. In order to formulate specific policies for screening migrants for infectious diseases, the national context needs to be taken into account—the epidemiology of diseases in each country (and in its specific migrant population), the health system framework, the priorities of health and social care for migrants—as well as the existing evidence on the effectiveness of screening, some of which is presented in this series.

Another shared theme in a number of the articles is the need to ensure migrants with a positive screening result have access to health care and treatment. Barriers to these are often present, and include structural and cultural aspects. Providing migrants with access to appropriate health care makes good public health sense, is a fundamental human right tied to the principle of non-discrimination and should be ensured by hosting countries as emphasised, for example, by the International Organization for Migration and World Health Organization [22]. Screening should never be seen as the application of ‘just a test’, but as a first step leading to diagnosis and treatment of those who are likely to benefit from it.

Screening for certain infectious diseases is important and, if appropriately implemented, can be cost-effective and contribute to the prevention of disease in migrants and their host communities in Europe. It is essential that the wider context affecting migrants is taken into consideration when implementing screening programmes. Optimally, screening should be part of comprehensive approaches that address all aspects of migrants’ health needs and vulnerabilities, and particular effort should be made towards this end [22].

## *Erratum

Due to an error, Afghanistan, Syria, Albania and Kosovo** were introduced as main countries of origin for individuals in the German study. This was corrected on 17 July 2018.

**Note**

**This designation is without prejudice to positions on status, and is in line with UNSCR 1244/1999 and the ICJ Opinion on the Kosovo declaration of independence. This note was added on 26 July 2018.
