# Erratum for Euro Surveill. 2018;23(28)

**DOI:** 10.2807/1560-7917.ES.2018.23.29.1807191

**Published:** 2018-07-19

**Authors:** Eurosurveillance editorial team

**Affiliations:** 1 European Centre for Disease Prevention and Control (ECDC), Stockholm, Sweden

In the article ‘Screening for infectious diseases in newly arrived migrants in Europe: the context matters’, published on 12 July 2018, Afghanistan, Syria, Albania and Kosovo were erroneously introduced as main countries of origin for individuals with positive stool results for *Schistosoma mansoni* eggs in a study from Germany. The mistake was corrected on 17 July 2018. 

